# Knowledge, Attitudes, and Perception as Predictors of COVID-19 Safety Practices of Ride-Hailing Operators in Ghana: A Cross-Sectional Study

**DOI:** 10.3390/ijerph20054529

**Published:** 2023-03-03

**Authors:** Ernest Agyemang, Joseph Awetori Yaro

**Affiliations:** Department of Geography & Resource Development, College of Humanities, University of Ghana, Accra P.O. Box LG 59, Ghana

**Keywords:** COVID-19, knowledge, attitudes, and practises (KAPs), health belief model, ride hailing operators, Accra, Kumasi, Ghana

## Abstract

Since its outbreak, health authorities have launched vigorous COVID-19 health promotion campaigns. This study assesses ride-hailing operators’ COVID-19 knowledge, attitudes, and practices in Ghana, with the aim of engendering precautionary behaviour among the populace. A complementary mixed methods approach was adopted. This involved a cross-sectional survey of 1014 participants who were also allowed to share their COVID-19-related lived experiences qualitatively after successfully completing the survey. The aggregate correct knowledge score was 84%. Most respondents were frightful of the virus (96%), but the majority had faith in the COVID-19 protocols (87%). Thus, most participants reported high use of face masks (95%) and practise personal hygiene (92%). However, social media misinformation and the subsequent complacency have dissuaded some participants from complying with the safety protocols. The qualitative data also show evidence of high susceptibility to COVID-19. The perceived benefits of safe behaviour, including masking up, were equally high among drivers surveyed, albeit barriers to preventative behaviours remain rife. Therefore, this study emphasises the importance of sustaining and improving public awareness by highlighting the susceptibility of all demographic groups to the virus and the need to counteract misinformation on social media.

## 1. Introduction

Owing to spatial interactions made possible by modern transportation systems, the deadly COVID-19 disease—which was first reported as ’a cluster of viral pneumonia’ in Wuhan, the provincial capital of Hubei in China, on 31 December 2019—quickly spread to worldwide [1,2]. Studies show that by 31 January 2022, a little above 2.9 million SARS-CoV-2 daily infections have been reported globally [3]. Recent studies even caution against the so-called “post-COVID-19 conditions”, “long COVID”, or “long-haul COVID”, including pulmonary, cardiovascular, neurological, and physical disorders which are emerging worldwide [4,5]. For instance, Ref. [5] estimates that nearly 1 in 13 US adults, either with or without a previous COVID-19 diagnosis, currently have post-COVID conditions. The possible causes of this trend are difficult to pinpoint. However, it cannot be overemphasised that as the pandemic continues, there is the need for extra vigilance, further scientific knowledge, and strategic public health campaigns to engender strict compliance to the COVID-19 protocols as were rolled out during the first wave of infections in 2020.

Ghana’s COVID-19 cases were first reported in Accra, the national capital, on 12 March 2020 [6]. By the end of March 2020, Ghana had confirmed 152 new locally transmitted cases, with a death toll of five victims, and a recorded 22 recoveries [7]. Consequently, several emergency response measures were introduced to minimise the communal spread of the virus. Table 1 summarises mitigation protocols explicitly mandated for public transport operators and users, as well as the issuing institutions of the state. For instance, the Parliament of Ghana promulgated Act 1012 to provide the legal basis for the Presidency to impose a lockdown order. The lockdown took effect in Accra and its contiguously built-up area, known as the Greater Accra Metropolitan Area (GAMA) and the Greater Kumasi Area (GKA) in the Ashanti region, between 29 March 2020 and 20 April 2020. Offenders of Act 1012 were liable for a summary conviction, a fine of between GHS 12,000 (USD 2068) and GHS 60,000 (USD 10,343), and a jail term of between 4 and 10 years. In addition, the Government of Ghana embarked on a massive public health campaign through traditional and social media outlets all over the country [8]. The safety campaigns provided ample information about the virus, its mode of spread, and mitigation measures. Additionally, various state and non-state actors donated sanitary items to help public transport operators in the fight against the pandemic. The Ministry of Transport, for instance, donated 200 Veronica Buckets (i.e., running water basins), 200 plastic bowls, 800 pieces of hand sanitisers, 400 pieces of Detol, 400 pieces of liquid soap, 400 pieces of tissue and 300 pen drives containing recorded messages on good hygiene. Two major ride-hailing companies, Uber and Bolt, were each given 40 pieces of hand sanitisers, 20 pieces of Detol and 20 pen drives [9].

Earlier exploratory observational studies assessed compliance with COVID-19 mitigation measures. In Accra, results show that hand washing at public transport stations was woefully infrequent, and in most instances, only a few passengers wore face masks [10]. Similarly, fewer public transport users wore face masks in Kumasi. Meanwhile, physical distancing measures were strictly enforced [11]. In these two major cities, the technology-enabled ride-hailing services operated by transportation network companies such as Uber, Bolt, and Yango, among others, are popular public modes of transport [12]. Public transport has been identified as a potential source of COVID-19 infection [10]. Proximate seating patterns in public transport have become fertile grounds for transmitting the infectious virus, especially as drivers and passengers come into direct or close contact with other people on board who may be asymptomatic COVID-19 carriers.

A knowledge, attitude, and practices (KAP) survey is defined as “a quantitative method (predefined questions formatted in standardised questionnaires) that provides access to quantitative and qualitative information” [13] (p. 1). These surveys generally measure peoples’ perceptions and opinions, which may serve as triggers or constraints on behavioural change. This paper focuses on the KAPs of ride-hailing operators in Ghana, given the urgency to develop effective public health and infection prevention measures to break the chains of transmission in the transport sector. This is because—despite the evolving knowledge of the science of transmission of the virus, by which the Government engages the public through several agencies and media houses—there is a huge lacuna in knowledge among the population, with reflections on safety practices. Belief systems and conspiracy theories abound both globally and in Ghana outside the established WHO science, which may produce a counter trajectory of behaviour among the population. Earlier KAP studies [14,15,16,17,18,19,20] have provided better scientific insights on developing preventive strategies and health promotion programs among the general population and among frontline health caregivers, college students, villagers, dental academics, and public hospital visitors. However, ride-hailing operators appear not to have received any particular focus so far in the scientific debates. Scientific evidence of drivers’ appreciation of the gravity of the pandemic and their compliance with the highly publicised COVID-19 protocols, in addition to their insistence on clients’ compliance, is an essential milestone for achieving the health and transport authorities’ targeted interventions seeking to curtail the communal spread of the virus.

Thus, the three-fold specific objectives of this paper are to assess: (1) COVID-19 knowledge levels among drivers, (2) their attitudes towards the pandemic, and (3) their recent practices deriving from the need to stay safe.

## 2. Conceptual Framing: The Health Belief Model

This study adopts aspects of the health belief model (HBM) as its analytical framework. The HBM conceptualises the health-related behaviours of individuals on the premise that humans generally want to avoid illnesses or desire to become well soon after they become ill. Thus, individuals will pursue specific health action that prevents or cures an infection. In other words, an individual’s belief in a personal threat of an illness or disease and the efficacy of a recommended health behaviour or action will predict the likelihood that that individual will adopt a proposed behaviour. An individual’s health behaviour is also a function of their health literacy status, defined as “the degree to which individuals have the capacity to obtain, process, and understand basic health information and services needed to make appropriate health decisions” [21]. Besides personal health literacy, the role of organisations (both local and international) in promoting health literacy and services, particularly during disease outbreaks, for example, COVID-19, is critical in achieving equitable health [22].

Within the context of the HBM, health behaviours and responses to proposed interventions are because of the interplay among factors such as a person’s perceived threat to sickness or disease (perceived susceptibility), the belief of consequence (perceived severity), and the potential positive benefits of action (perceived benefits). The rest are perceived barriers to action, exposure to factors that prompt action (cues to action), and confidence in the ability to succeed (self-efficacy). Additionally, the socio-demographic profile of an individual and psychological factors, including their personality and pressure from their significant others, are known to influence health-related behaviour. This paper adopted the HBM as an explanatory framework due to its extensive application in recent studies that explain preventative behaviours. They include positive behaviours against malaria among Filipino migrant workers and Taiwanese returnee migrants [23,24], as well as against dengue fever among Malaysian high school students [25] and COVID-19 vaccination outcomes among the general Pakistani population [26]. These studies unanimously found that participants’ improvement of knowledge, perceived severity, benefits, and barriers directly impacted positive preventative behaviour and good health outcomes. To illustrate, for instance, a study found that Saudis who perceived the severity of respiratory infections to be high responded positively to health campaigns to regularly sanitise their hands and mask up when sneezing or coughing [27]. A recent study has also adopted the HBM to gauge and explain the compliance of informal paratransit operators to the masking up protocols in Ghana. Like the earlier studies, older drivers who perceived their vulnerability to COVID-19 to be high consistently complied with face mask use [8].

In light of the studies mentioned above, this present paper proceeds with the hypothesis that:

There is a statistically significant difference in COVID-19 safety practices among the demographic characteristics of participants in the study.

## 3. Materials and Methods

### 3.1. Study Sites and Population

**Figure 1:**
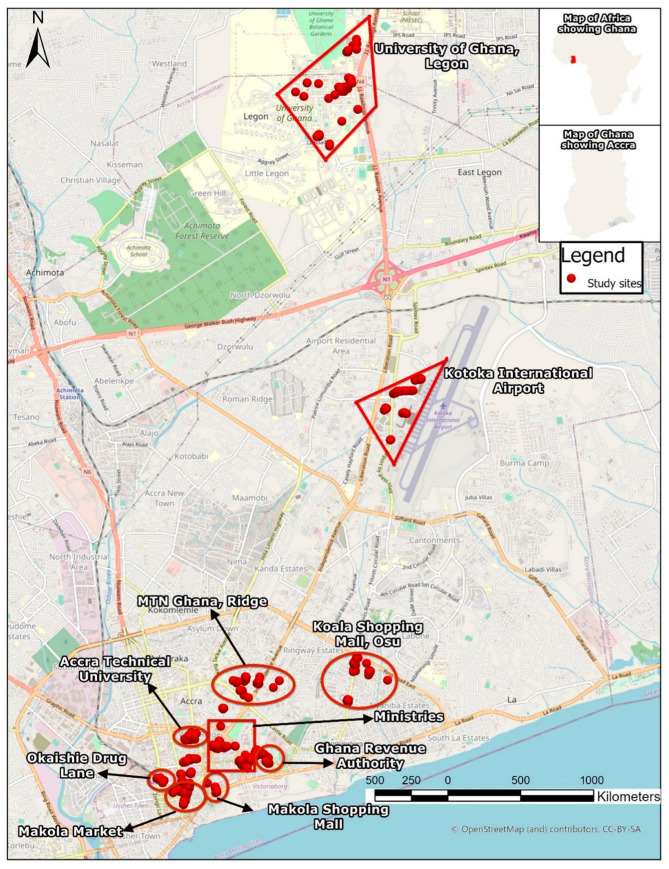
An annotated map of study sites in Accra.

**Figure 2:**
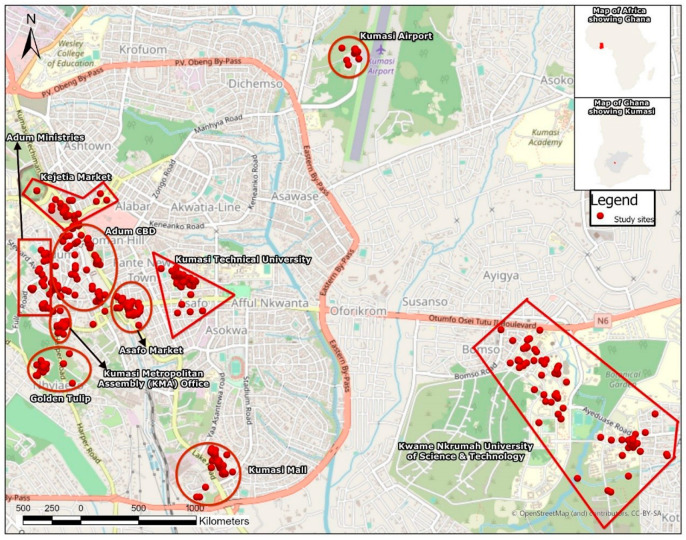
An annotated map of study sites in Kumasi.

The overarching objective of this paper is to measure the knowledge, attitudes, and practices of ride-hailing operators in Accra, Ghana’s national capital and home to an estimated population of over 5 million, as well as in Kumasi, Ghana’s second most urbanised area with an estimated population size of over 3 million [28]. To achieve this objective, a cross-sectional design was used to generate quantitative data largely through a self-administered questionnaire survey deployed on the KoBoToolbox platform. The various study sites are illustrated in Figure 1 and Figure 2.

### 3.2. Research Design

This study adopted a complementary mixed-methods approach involving quantitative and qualitative data collection techniques.

#### 3.2.1. Quantitative Data

Regarding the quantitative data, a multi-stage systematic sampling technique was adopted in selecting study sites and respondents. At the first stage of the sampling, all locations designated for higher education, formal commercial centres such as shopping malls and supermarkets, telecommunication offices, government/public offices, and airports, where ride-hailing operators are most likely to concentrate their services were specifically extracted from Google Maps and imported into an Excel spreadsheet. The application of Google Maps proved very useful in the sense that this mapping and consumer-based web application allows users of smartphones—one of the basic tools for ordering ride-hailing services—to review and rate locations and businesses they have visited. High user ratings may suggest that such locations are very popular with netizens. Thus, at stage two of the sampling procedure, sixteen (16) highly rated specific sites where various formal sector jobs/offices and educational institutions are located were selected. These included tertiary institutions, shopping malls, supermarkets, telecommunication offices, government/public offices, and airports where formal sector employees and students—who are the primary users of ride-hailing services—usually visit. Stage three included the purposive sampling of popular markets and informal commercial centres such as Okaishie Druglane and the Makola Market (in Accra), as well as the Asafo and Kejetia Markets (in Kumasi), as study sites. From our experience, these informal geographic spaces are known to attract a large number of trip-makers to the central business districts of these two major cities to engage in informal commercial activities. Thus, it was reasonable to expect to locate ride hailing operators in these locations as well. However, these informal spaces seldom received mentions and reviews on Google Maps. Therefore, we assumed that their diminished presence on Google Maps might not imply less patronage. Instead, most trip makers in the informal sector do not necessarily use these ‘high-tech’ application tools such as Google Maps. The fourth and final stage of the sampling involved the random selection of study participants at the study sites. The surveys were conducted simultaneously in both cities from 25 January to 4 March 2021. Research assistants installed the ride-hailing apps Uber, Bolt, and Yango and randomly booked rides. Once the vehicle arrived, the assistants politely introduced the survey to the drivers, and consenting drivers were paid for the booking. The trip was then cancelled on the apps for the interviews to commence. This was how drivers were invited to participate in the study in most instances. This approach was adopted because, during a pilot of the survey, most participants abruptly abandoned the survey as soon as riders ordered their service. At other times, the assistants took notice of the major rest stops where most ride-hailing drivers took their breaks and met to chit-chat with each other. They were invited to participate in the survey, and willing participants volunteered information without demanding any form of compensation. Eligible participants were supposed to respond ‘Yes’ to the question: “As an Uber, Bolt, or Yango partner driver, have you driven at least 4 rides?” to be included in the survey. In all, one thousand and fourteen (1014) respondents took part in the survey.

In the design of the survey instrument, we took inspiration from KAP measures developed in two previous studies, which were conducted in Malaysia and China [15,29]. The questionnaire was sectioned into four parts, including the socio-demographic details of participants, such as gender, age, education, marital status, ride-hailing type, and city of residence. In addition, questions bordering on participants’ knowledge about the COVID-19 pandemic, as well as general attitudes and practices occasioned by COVID-19, were asked. In measuring participants’ knowledge about the disease, 13 items were adapted from the previous foreign studies indicated and were complemented with additional 5 specific items related to Ghana’s enhanced COVID-19 mitigation measures for public transport (see [8]). The knowledge items measured included COVID-19 clinical symptoms (items 1–3), origin and mode of transmission (items 4–7), as well as prevention and mitigation of COVID-19 (items 8–18). For each item, participants were expected to provide answers from the options “True”, “False”, or “No Idea”. One mark was given to each correctly responded answer, while all wrong and unsure responses were assigned 0 points. The maximum total score a participant can obtain ranged from 0–18, with a higher score indicating that one had ample knowledge about the COVID-19 pandemic.

On ‘attitudes’, respondents were queried on whether they were worried about rising COVID-19 cases, with answer options ranging from “Yes, absolutely” and “Yes, but not as it was in March 2020” to “No”. In addition, respondents were asked, “do you perceive yourself to be vulnerable to COVID-19?” and “do you have confidence in the government’s mitigation measures against COVID-19?”. Respondents were provided ‘Yes’ or ‘No’ responses to choose from.

Regarding ‘practices’, participants were asked to indicate whether they had visited crowded public places in the most recent past, travelled in a public means of transport, properly washed and sanitised their hands, and used a face mask when leaving home. The answer options were either ‘Yes’ or ‘No’. The questions were presented to the participants in either English, the colloquial street language ‘Pidgin’, or Asante, Twi depending on the participants’ preferences.

The raw quantitative data were exported from the KoBoToolbox platform into an Excel spreadsheet and cleaned to improve data quality. Data were coded and analysed using the Statistical Package for Social Sciences (SPSS) 25 software. Preliminary treatment of the data regarding consistency and reliability resulted in a score of 0.72 on the Cronbach’s Alpha reliability co-efficiency scale, implying that the data are robust, consistent, and reliable [15,30]. Descriptive statistical analyses were later performed, including frequencies and percentages. The influence of selected socio-economic and demographic independent variables on the dependent variables (knowledge, attitude, and practices) was regressed using the analysis of variance (ANOVA) statistical tool. Additionally, statistically significant differences among the constructs were assessed using Pearson’s chi-square test.

#### 3.2.2. Qualitative Interviews

Short qualitative interviews were also conducted to allow respondents to present their lived experiences in their own words. We adopted the qualitative method to explain and reinforce aspects of the quantitative findings, and to explore deeper meanings of stated behaviour [31]. Survey participants were required to answer either “yes” or “no” to the question: “Personally, do you think you are vulnerable to contracting the COVID-19 virus as you regularly get in touch with strangers who board your car?” All who responded in the affirmative were further required to “explain why you think you’re vulnerable to COVID-19 infection”. This allowed participants to share their COVID-19 experiences in their own words. Several reasons were recorded verbatim and typed as text on the field. In all, 4218 words were extracted from the typed voices of the survey participants in response to the question. In “winnowing” the data before analysis, as pointed out by [32,33], we used the “Find and Replace” command in Microsoft Excel to reclassify all synonymous words into more familiar words. For instance, words such as “people”, “other people”, “other persons” and “strangers” that appeared in the data were all replaced with the word “passengers”. Additionally, words such as “cash”, “money”, and “fare” were replaced with the word “cash”. The qualitative data were then imported into Microsoft Word and analysed for frequently occurring words using a Pro Word Cloud generator (Version 1.0.0.3). We set the maximum words to 50 and further instructed the software to remove all common words. In all, 395 frequently occurring words were selected. For instance, the word “passengers” was used 315 times, “cash” appeared 107, “different” was used 96 times by participants. The output was then visualised as word cloud and presented as a figure. The size of each word demonstrates how important it was to survey participants in explaining why they felt vulnerable to COVID-19 infection. Studies have shown that word clouds provide novel and reader-friendly approaches for analysing and presenting qualitative data [34,35].

Further, we allowed respondents to share their attitudes and general thoughts on the pandemic qualitatively. The researchers saved the interviews using voice recorders on their smartphones. The interviews lasted an average of ten minutes per participant. Voice recordings of selected survey participants were played back and transcribed verbatim by the first author. The researchers translated directly into English for participants who spoke in the local dialects. The first author thoroughly read, highlighted, and annotated relevant sections of the transcripts. Next, the second author independently combed through, highlighted, and categorised all statements, sentences, or quotes from the raw qualitative data. Both authors then debated and developed clusters or categories relative to the HBM constructs, to shed light on how participants perceived their vulnerability to and coping mechanisms to prevent COVID-19 infection. This inductive coding was performed thoroughly until all categories in the data had been exhausted. The coding categories include “perceived susceptibility”, “perceived severity”, “perceived benefits”, and “perceived barriers”. The researchers treated each statement as having equal worth. We then adopted the textual description approach and presented participants’ COVID-19-related lived experiences as direct quotes in the study.

To ensure the data’s credibility, and following previous studies, we employed the member-checking strategy by having some participants check the transcribed data for accuracy and clarification of information. As has been conducted elsewhere [36,37], we made contacts through phone calls to some of the participants to substantiate some of the direct quotes we had translated from the interviews to maintain the participants’ meaning, and ensure trustworthiness, authenticity, and credibility, as cautioned by [32].

## 4. Results

### 4.1. Demographic Characteristics of Participants

The data are skewed disproportionately towards males, who constitute 99.7% of the total 1014 participants in the survey. Few women operate ride-hailing or even drive public transport in Ghana, hence resulting in their small share in the dataset. The average age of participants was 32 years (SD = 5.7, range = 20–69). The participants had received at least 15 years of formal education (SD = 2.9, range = 0–20), and the majority (46.6%) had a senior secondary school certificate. Most drivers (76.5%) indicated that they mainly operate for the Bolt ride-hailing company. Regarding driving experiences as ride-hailing operators, the average period was 27 months (SD = 17.3, range = 1–62). Table 2 summarises the rest of the background information on survey participants.

### 4.2. Assessment of Knowledge

Of the 18 items that were used to measure the participants’ knowledge about COVID-19, the average score recorded was 15.1 (SD = 2.0, range = 0–18). The correct aggregate score on the knowledge measures was 84% (15.1/18 × 100). About 78.9% of participants scored 15 and above, indicating an acceptably high level of knowledge of COVID-19.

Regarding knowledge of the clinical manifestations of the disease, most participants (96.3%) knew that COVID-19 patients usually have symptoms such as fever, runny nose, dry cough, sore throat, and headaches. On the origins and transmission items, many respondents (53.4%) incorrectly thought that the virus could be transmitted through the consumption or handling of game. Despite this, 96.9% and 86.8%, respectively, of participants correctly said that the COVID-19 virus spread through respiratory droplets and contact routes as well as airborne transmissions.

On how we can best control and mitigate transmissions, most participants correctly said there was the need to practice frequent hand hygiene (97.6%) before touching eyes, nose, and mouth, as well as exercising respiratory etiquettes of covering the nose and mouth when coughing or sneezing (97.8%). Regarding the specific enhanced mitigation measures announced by the Government of Ghana for public transport operators, the order on face mask use (96.9%), and the practising of physical distancing in vehicles (97.5%) were the most widely known measures that most participants scored correctly. On the downside, as many as 60.4% of participants scored incorrectly on the Government’s directive for drivers to write down the contact details of all passengers for possible contact tracing should the need arise. See Table 3 for a summary of the other findings.

### 4.3. Assessment of Knowledge

As many as 96% of the participants indicated that they were anxious about the COVID-19 pandemic when asked whether they were worried or not about rising cases during Ghana’s second wave of infections in early 2021, as explained in the qualitative interviews, as follows:

“Indeed we were extremely worried at the beginning of this year [2021] when we heard that new active cases were emerging. Some of us thought that the worse was over in 2020, but then we woke up only to realise that the disease had come again [resurfaced]”.

(A 23-year-old Uber driver in Kumasi)

“I think the Christmas and elections period [2020 General Elections in Ghana] took away our attention from the fear we had from the beginning of last year. So it is right now that the excitement is over that we are waking up to the frightening realities”.

(A 25-year-old Bolt driver in Accra)

**Figure 3:**
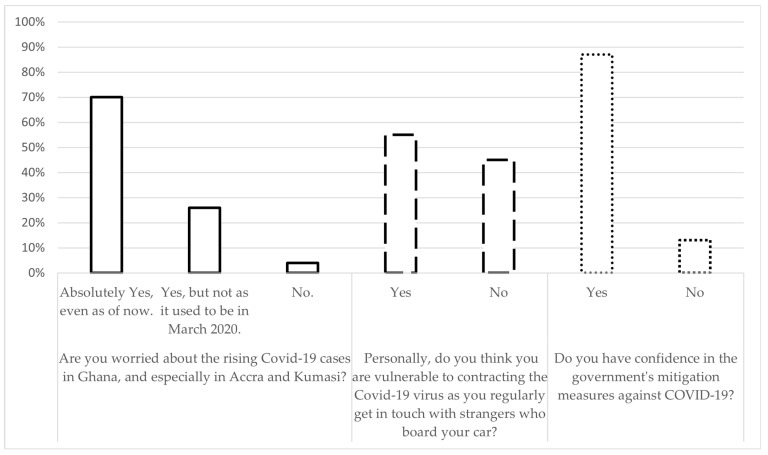
Attitudes of participants regarding COVID-19 (N = 1014).

As shown in Figure 3, a majority (70%) of participants who expressed fear about the rising COVID-19 cases, however, felt that their level of anxiety was higher during the first wave of infections in Ghana, sometime in March 2020, compared to the second wave (26%). Most ride-hailing drivers (55%) felt they were vulnerable to quickly catching the COVID-19 virus. This notwithstanding, most of the respondents (87%) believed in the Government’s COVID-19 management and were optimistic that the nation would ultimately overcome the pandemic.

The qualitative data reveal what seems to the source of the heightened anxiety among survey participants. The drivers asserted that their work exposes them to passengers from different backgrounds whose COVID-19 and vaccination status may not be known to them. Additionally, the exchange of physical cash at the end of trips, instead of digital payment options, predisposes the drivers to the threat of COVID-19, as seen in the following comments:

“I strongly agree that we drivers are very much at a higher risk of catching the virus. No matter how cautious you may be, you are always picking up passengers who may be carriers of the disease. Some carriers may themselves be asymptomatic, and before you’re aware, you’re already sick and all that”.

(A 27-year-old Uber driver in Kumasi)

“Presently, I haven’t taken the shot, and I also do not have any means to confirm the COVID-19 health status of the passengers whom I serve. So, yes, I think commercial drivers are as exposed as the frontline health care workers, or even worse”.

(A 30-year-old Bolt driver in Accra)

“I say we [drivers] are exposed to the disease because, see, we take money from passengers each day. So if the surface of money is contaminated, how will you know? That’s why we constantly sanitise our hands. Sometimes too, some passengers are difficult people, and if you insist too much that they comply with the protocols, they can ignore you or, worse, refuse to join your car any longer”.

(A 32-year-old Yango driver in Accra)

**Figure 4:**
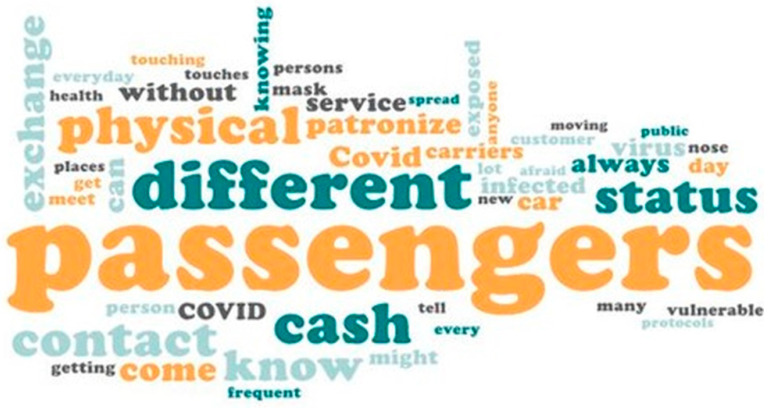
Key factors heightening drivers’ perception of vulnerability.

Figure 4 further illustrates the key factors which appear to increase the drivers’ level of anxiety towards catching the COVID-19 virus.

As shown in Table 4, when the data are split into socio-demographic and spatial categories, all the independent variables (i.e., age, marital status, and education) scored more than 80% in positive attitudes, with no major statistically significant variations.

The qualitative interviews shed light on optimistic attitudes toward the COVID-19 mitigation efforts. The open and transparent manner by which the Government regularly updated the citizens and the role of faith-based organisations in providing support to its members in times of adversity helped considerably. In addition, media awareness of the public may be a critical determinant of the hopeful attitudes, as seen in the comments below:

“I believe that we are on course to win the battle against the virus. I believe in the President [of the country] and his regular assurances that ‘this one too shall come to pass’ anytime he comes to update the nation on measures he and his administration are putting up to keep us safe”.

(A 32-year-old Yango driver in Accra)

“For me, my faith in the Lord Almighty tells me that certainly, this devilish pandemic will be overcome. At worse, it [COVID-19] will become normal, like malaria and other diseases that we constantly live with”.

(A 39-year-old Bolt driver in Accra)

“I agree that there has been massive sensitisation in the media. Initially, they were only reporting the case count and deaths here in Ghana, but when you watch foreign news outlets and you see the plight of Corona sufferers in hospitals and also the mass burials of victims, I thought the end of the world had come. But at least for now, it has been a year, and we are still alive. We give thanks to God, and I’m convinced that we shall overcome”.

(A 28-year-old Uber driver in Kumasi)

### 4.4. Assessment of Recent Practises

**Figure 5:**
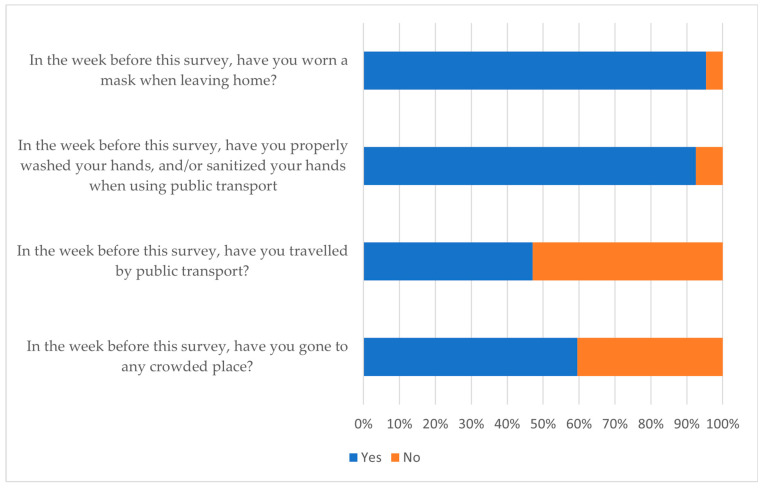
Practises of participants regarding COVID-19 (N = 1014).

Participants were quizzed on their most current practices, such as visiting public places, using public transport, maintaining hand hygiene, and using a face mask during the week preceding the conduct of the field survey. While most participants had recently visited public spaces and used commercial transport, most wore face masks (95%) and ensured that proper hygiene was observed (92%) to stay safe. Figure 5 illustrate the various responses.

An interesting pattern that emerges in the data is that despite socio-economic and demographic differences among participants, compliance to face masking and proper hygiene—two of the most potent mitigation measures—were all above 80%.

Table 5 further shows the results of a chi-square test with regard to the study hypothesis. The study’s hypothesis was rejected given that the results did not show any statistically significant variations among respondents in washing or sanitising hands. Persons who had obtained second cycle and tertiary education reported less use of public transport. The wearing of facemask was high among all the various groups, except respondents who had only completed basic school.

Facemask use, in particular, was found to be statistically related to the perception of being vulnerable, as illustrated in Table 6. Out of the 967 participants who reportedly wore their facemasks, the data show that they did so because they felt that they were at serious risk of receiving the virus (55.9%).

In addition, findings from the qualitative interviews on participants’ COVID-19 perceptions, extracted through the analytical lenses of the health belief model, are summarised in Table 7.

The key themes emerged from the dataset border on participants’ perceived susceptibility to COVID-19, the severity of sickness, the benefits of safe behaviour, and barriers to the adoption of mitigation protocols. These are also highlighted in the sentiments expressed by participants, as follows:

“I consistently wear my facemask. I don’t joke with it. With all that is happening around us and the way people are dying, no one should advise you to take precautions. Especially for some of us who are always in constant contact with strangers [passengers], I don’t think you should allow the government to force you to stay safe”.

(A 39-year-old Uber driver in Accra)

“As I said earlier on, we drivers are vulnerable given the nature of our profession. So, for me, it’s safety first, and besides, it’s mandatory, per our company’s rules, to always mask up, whether as passengers or drivers”.

(A 27-year-old Uber driver in Kumasi)

“Nowadays, I don’t really wear a face mask, to be honest with you. For some time now, we [drivers] hang around to chat and even eat together during breaks. So far, none of my colleagues I know has been found to have contracted the disease, so I don’t think there is the need to worry ourselves any longer”.

(A 28-year-old Uber driver in Kumasi)

“It’s true that we may all be vulnerable, but if you’ll fall sick or not, it’s not the facemask that can save you. The disease only affects the aged, and as you can see, we are young people. Moreover, it is challenging and uncomfortable to breathe while wearing a facemask. Personally, if a passenger comes on board already masked up, why should I bother myself to wear the facemask again?”

(A 20-year-old Uber driver in Kumasi)

The role of the internet in causing some people to become misinformed and subsequently refuse to adhere to the COVID-19 protocols, including masking up, was also clearly apparent in the qualitative data:

“I actually don’t think wearing the facemask is the remedy to catching the virus. My point is that the scientific evidence I have seen suggests that it’s only persons who have underlying conditions who are the most susceptible to the virus.

So I think people should rather focus on maintaining a stronger immune system through regular exercise and a good diet”.

(A 26-year-old Bolt driver in Kumasi)

“I think there are better ways of taking care of one’s self. At least on social media, one can find useful videos and educational materials on how to stay fit and avoid the virus. Did you know that by inhaling steam from boiled neem leaves [Azadirachta indica], all the viruses can die off even if you become sick? Check the internet; it’s all there. As a matter of fact, I think this is a hoax, and very soon, the truth behind this will come out”.

(A 30-year-old Bolt driver in Accra)

## 5. Discussion

Following the surge in COVID-19 infections in Ghana in March of 2020, the government enforced a stay-at-home order for non-essential trip makers in the two largest cities of Accra and Kumasi. The government cautiously lifted the month-long mobility restrictions and announced several enhanced mitigation measures specifically targeting public transport operators, such as ride-hailing drivers and their passengers. Ghana, like many other African countries, was hailed for its effective management of the pandemic [38]. In what appears to be a lack of sustained effort to contain the virus, Ghana’s COVID-19 enviable record was put to the test when, on 2 February 2021, official sources cautioned that some 772 new cases had been detected, thus raising the total active COVID-19 cases to 5515. In addition, eight new deaths due to COVID-19 complications were reported, therefore increasing the death toll to 424 [39,40,41]. Against this backdrop, this paper specifically sought to assess (1) COVID-19 knowledge levels among drivers, (2) their attitudes towards the pandemic, and (3) their recent practices deriving from the need to stay safe.

A major finding made in this paper relates to the high aggregate correct scores obtained by participants on the COVID-19 knowledge measures. With about 84% of participants being knowledgeable about the disease, the implication is that information dissemination has gone down well among surveyed participants. Irrespective of the differences in measurement and scoring systems used in previous studies, the overall correct answer rate on COVID-19 knowledge among Ghanaian ride-hailing operators is appreciably higher than scores reported in earlier KAP studies, including among healthcare providers in southwest Ethiopia, who scored 73.8% [19], and among Malaysians who scored 80.5% [15] but certainly lower compared to the 88% and 90% scores obtained, respectively, among Vietnamese health care workers in Ho Chi Minh City [42] and Hubei residents in China [29]. Most participants who scored higher on the COVID-19 knowledge test in Ghana were between 18–39, never married, and lived in Accra, the national capital. This finding corroborates an earlier study that found higher knowledge scores among young people (aged 18–29 years) than other age groups in Nigeria and Egypt [43]. However, it contradicts other studies that reported higher knowledge scores for older Bangladeshi and Malaysian people (aged 45 years and older) than for younger participants [15,44].

In a recent study [8], the extensive media coverage of the pandemic, as well as regular televised COVID-19 updates and personal pleas from the Ghanaian President, were found to be explanatory factors for the high knowledge of COVID-19 in the country. In addition, most of the transport network companies displayed relevant COVID-19 mitigation information on their apps for the benefit of both drivers and passengers on the requirement to wear facemask over the mouth and nose while riding, to always sit in the back seat (for passengers), and also to keep the windows open for proper ventilation. It may explain why most drivers were highly knowledgeable about the pandemic. However, while higher knowledge scores were generally reported, some ignorance was observed in the data. This is particularly in connection with the origins and transmission questions. As many as 53.4% of participants incorrectly thought that the virus could be transmitted through the consumption or handling of bushmeat.

This may partly be explained by respondents’ prior knowledge of the mode of transmission of the Western African Ebola Virus epidemic (2013–2016), which was believed to have evolved from the consumption of bats, gorillas, and duikers.

Again, as the pandemic unfolds and scientific knowledge also grows, sometimes it may generate some confusion among the populace as to which information is correct or otherwise. For example, as of 29 March 2020, the World Health Organization (WHO) had publicly identified two critical sources for the transmission of the COVID-19 virus. These were transmission between people through respiratory droplets and contact routes. The theory of airborne transmission was rejected [45]. Four months later, and based on new evidence, the WHO admitted airborne transmissions, together with fomites (contaminated surfaces) as some possible routes of infection. Specifically, the airborne transmission was said to occur in indoor and crowded spaces [46]. Thus, it is imperative that as new and better knowledge about the disease is reported, extra efforts must be put in place to re-educate ride-hailing operators, in particular, and the public at large, to avoid all sorts of confusion.

The study further found that 60.4% of surveyed participants scored incorrectly on the Government’s directive for drivers to write down the contact details of all passengers for possible contact tracing. While this was a laudable strategy, it seems its implementation was fraught with serious challenges, especially for intra-city commercial transport operations whereby passengers usually travel over shorter distances. Thus, this directive was mainly ignored, and over time, most participants forgot that such a directive even existed. Perhaps policymakers can take a cue from this and make sure to roll out only pragmatic strategies which are also enforceable for effective management of the pandemic. A clear case was the Government’s outright failure to enforce Act 1012 strictly, which was possibly due to its harshness and the fear of potential backlash from voters, especially given that the law was passed in the run-up to the 2020 general elections. To ensure enforceability, authorities may have to amend the law and introduce realistic fines and other community services to deter COVID-19 offenders.

Concerning participants’ attitudes towards COVID-19, most surveyed participants were very frightened about the disease during the first wave when people were less knowledgeable about the symptoms, risk factors, and mitigation measures. During Ghana’s second wave of infections in early 2021, however, the level of anxiety had reduced considerably. The implication could be positive in that perhaps, the public heeded the pieces of advice not to panic but to comply with the enhanced mitigation measures strictly. On the other hand, the reduced level of stress about the disease could also imply complacency or outright rejection that the pandemic exists. The negative implications of this finding for COVID-19 case management cannot be overemphasised.

Using the HBM analytical framework, some notable observations were made from the qualitative data regarding health beliefs and perceptions of vulnerability to COVID-19. While many ride-hailing drivers had high perceived susceptibility to becoming severely ill with COVID-19, particularly during Ghana’s second wave of infections, there were some notable perceived barriers to preventative behaviours. These include the fact that some colleague drivers were still healthy and had never fallen ill with COVID-19, and participants perceived themselves to be without any co-morbidities. This suggests that it is imperative to increase risk perception among drivers, as high-risk perception has been found in earlier studies to promote preventive actions, including the willingness to become vaccinated against COVID-19 [47] and other influenza-like illnesses [48,49]. If this is not conducted, as found elsewhere, reduced susceptibility perceptions among the populace usually lead to complacency and adversely affect preventive behaviour [50,51].

Thankfully, as noted in the study, most of the participants have high perceptions of the benefits in preventative behaviours such as wearing the facemask, which, among other benefits, makes them feel safe and makes them keep their driving professions, as employers were insistent on all employees masking up.

In addition, a majority of drivers agreed that they were vulnerable to infections due to the numerous interactions with passengers and the minimal use of digital payment options. On the latter point, a recent study [52] found that the virus does not survive at high levels for very long on banknotes. A few hours after infection, even at high doses, the levels and associated risk of infection appear low. Therefore, the health authorities need to provide adequate and accurate information to the public to dispel all forms of misinformation and disinformation, which are critical in building trust and confidence in the management of the pandemic. As found elsewhere, misinformation and disinformation can negatively affect people’s responses to health interventions [53,54].

So far, the evidence suggests that most drivers have positive attitudes toward the COVID-19 management strategies and are optimistic that the nation will ultimately overcome the pandemic, similar to a previous study [15]. This finding implies that most people’s trust the information being provided for its consistency and accuracy and are most likely to comply for their safety and that of their loved ones. This has been found in a recent Brazilian study which concluded that confidence in the capability of social institutions significantly translates into the adoption or otherwise of recommended protective behaviours [55].

The study also found that drivers who reported optimistic attitudes and perceived themselves as at high risk of infection (55.9%) reported wearing face masks consistently and practising proper hand hygiene. Unfortunately, as found in this present study, some participants who agreed that their profession significantly exposed them to the outbreak surprisingly indicated that they never consistently wore face masks when they went outdoors. This finding lends credence to an earlier Ghanaian study among commercial drivers [8], which reported that most drivers wore face masks, and there were others who reportedly never wore face masks. This is because the virus no longer posed any significant threat to their lives, and that face mask use was associated with discomfort. Finally, they were spiritually fortified against infection. Against this backdrop, this study presents yet another disturbing piece of evidence that should arouse the interest of relevant policymakers to intensify the ongoing public health education, especially among drivers.

As in all studies, this present study must be read with some limitations in mind. At the time of conducting the survey, the research instrument which specifically measured the KAP of participants was not designed using the health belief model questionnaire format.

Instead, the philosophical underpinnings of the HBM were adopted as an appealing analytical framework for making sense of the qualitative dataset. Future studies may take a cue from earlier studies [47,48,49] which adopted the HBM questionnaire format to frame the questions before the field investigations.

## 6. Conclusions

We assessed the knowledge, attitudes, and practices of ride-hailing operators in Ghana regarding the ongoing COVID-19 pandemic. We found that knowledge levels about the disease are relatively high among surveyed drivers. Most of them have a hopeful attitude towards the mitigation measures adopted to curtail community spread. Global and national efforts to sensitise people and enforcing rules and regulations have played a significant role in producing the positive outcomes shown by this study. The role of the media has been critical in both educating and instilling fear in people [20].

Given that public transport could promote the spread of the disease, it is understandable why efforts and achievements have been higher in this area. Persuasion alone is not enough to cause people to change their attitudes during pandemics, as shown in the Ghanaian case where police, military, and local government security personnel physically sanctioned offenders of protocols. Certainly, socio-demographic variables are essential in understanding differences in the uptake of information and instructions, changing attitudes, and influencing others. However, in pandemic situations, the state’s role in creating this awareness and enforcing new norms is paramount.

Therefore, weak states with poor capacities may achieve less in imparting new knowledge and engendering the uptake of appropriate practices through the enforcement of laid down protocols.

Given that full compliance to the established mitigation measures is yet to be achieved, the authorities need to pursue targeted interventions and health campaigns to further enhance knowledge about the pandemic to engender preventive behaviour among the populace.

## Figures and Tables

**Table 1:** COVID-19 mitigation protocols for public transport operation.

Mitigation Protocols	Issuing Authority
Enactment of Imposition of Restrictions Act 2020 (Act 1012)	The Parliament
Lockdown of Accra and Kumasi	The Presidency
Easing of lockdown and announcement of enhanced mitigation measures against COVID-19	The Presidency
Physical/social distancing in vehicles, e.g., taxis/ride-hailing services to reduce the total number of passengers from four to three per trip	Ministry of Transport
Transport terminals to ensure adequate running water basins and sanitisers for the use of passengers before boarding	Ministry of Transport
Commercial drivers to record contact details of commuters to ensure contact tracing when necessary	Ministry of Transport
Commercial drivers to thoroughly wash and disinfect vehicles after each trip	Ministry of Transport
Enforcement of mandatory wearing of face masks by commuters	Greater Accra Regional Coordinating Council
Education/sensitisation on face mask-wearing, social/physical distancing by the various MMDAs (i.e., local authorities) in their jurisdiction	Greater Accra Regional Coordinating Council
Commercial drivers to ensure all passengers wear face masks before being allowed to board their vehicles	Greater Accra Regional Coordinating Council
Notices of “No face mask, No entry” to be visibly displayed at vantage points, including offices, lorry stations, and shops, among others	Greater Accra Regional Coordinating Council

**Table 2:** Profile of survey participants.

Variable	Categories	Frequency	Percentage (%)
Gender	Male	1011	99.7
	Female	3	0.3
Age	18–29	440	43.4
	30–39	487	48.0
	40 and above	87	8.6
Education	No formal Education	12	1.2
	Primary (basic level)	18	1.8
	Junior High (basic level)	104	10.3
	Senior High (secondary level)	473	46.6
	Vocational/Technical/Commercial (secondary level)	98	9.7
	Tertiary (Bachelor’s and post graduate degree level)	309	30.5
Marital status	Never married	536	52.9
	Married	403	39.7
	Co-habiting	75	7.4
Ride hailing operator type	Bolt	776	76.5
	Uber	217	21.4
	Yango	21	2.1
Residential location	Accra	507	50.0
	Kumasi	507	50.0

**Table 3:** Knowledge of COVID-19 among ride-hailing operators.

Statements on COVID-19	Responses		
	True	False	No Idea/I Don’t Know
1. The main clinical symptoms of COVID-19 are fever, runny nose, dry cough, sore throat, and headache.	**976 (96.3)** *	27 (2.7)	11 (1.1)
2. Not all people with COVID-19 will develop to severe cases. Only those who are elderly, have chronic illnesses, and are obese are more likely to be severe cases.	**961 (94.8)**	35 (3.5)	18 (1.8)
3. There is currently no vaccine for COVID-19 in Ghana, but early symptomatic and supportive treatment can help most patients recover from the infection.	**933 (92.0)**	38 (3.7)	43 (4.2)
4. COVID-19 Viruses originated from animals to humans	**795 (78.4)**	4 (0.4)	215 (21.2)
5. COVID-19 Viruses can spread from human to human through cough, sneeze, and handshake	**983 (96.9)**	22 (2.2)	9 (0.9)
6. The COVID-19 virus is airborne	**880 (86.8)**	73 (7.2)	61 (6.0)
7. Eating bush meat or contacting wild animals would result in infection by the COVID-19 virus	343 (33.8)	**472 (46.5)**	199 (19.6)
8. To reduce rates of infections, you should wash your hands with soap and running water (or use sanitisers) before touching anything including your eyes, nose, and mouth.	**990 (97.6)**	17 (1.7)	7 (0.7)
9. To reduce rates of infections, you should cover your mouth and nose when you cough or sneeze.	**992 (97.8)**	15 (1.5)	7 (0.7)
10. Isolation and treatment of people who are infected with the COVID-19 virus are effective ways to reduce the spread of the virus	**989 (97.5)**	17 (1.7)	8 (0.8)
11. People who have contact with someone infected with the COVID-19 virus should be immediately isolated in a proper place. In general, the observation period is 14 days	**985 (97.1)**	20 (2.0)	9 (0.9)
12. To prevent the infection by COVID-19, individuals should avoid going to crowded places such as lorry stations and avoid taking public transportation	**905 (89.3)**	97 (9.6)	12 (1.2)
13. Since vehicles can be hotspots for COVID-19 spread, only people who really need to commute were permitted to do so. The rest were ordered to stay home	**985 (97.1)**	22 (2.2)	7 (0.7)
14. To prevent the rapid spread of COVID-19, public transport was obliged to practice social distancing, i.e., reduce the number of passengers on a row	**989 (97.5)**	19 (1.9)	6 (0.6)
15. To prevent the rapid spread of COVID-19, public transport operators and users were compelled to wear facemask	**983 (96.9)**	23 (2.3)	8 (0.8)
16. To prevent the rapid spread of COVID-19, public transport operators were urged to insist on a “no-facemask, no entry” policy for all passengers	**971 (95.8)**	20 (2.0)	23 (2.3)
17. To prevent the rapid spread of COVID-19, public transport operators were urged to insist on proper handwashing and sanitising for all passengers	**978 (96.4)**	0 (0.0)	36 (3.6)
18. To prevent the rapid spread of COVID-19, public transport operators were urged to collect the contact details of all passengers	**402 (39.6)**	376 (37.1)	236 (23.3)

* NB: Correct responses are indicated in bold. The figures in brackets are in percentages.

**Table 4:** Attitudes of participants by socio-demographic details.

Variable	Categories	Ghana Can Successfully Control COVID-19		Confidence in the Government’s Measures	
		Yes	No	Yes	No
Age	18–29	390 (88.6%)	50 (11.4%)	386 (87.7%)	54 (12.3%)
	30–39	419 (86%)	68 (14%)	419 (86%)	68 (14%)
	40 and above	77 (88.5%)	10 (11.5%)	76 (87.4%)	11 (12.6%)
Marital status	Never married	481 (89.7%)	55 (10.3%)	466 (86.9%)	70 (13.1%)
	Married	339 (84.1%)	64 (15.9%)	345 (85.6%)	58 (14.4%)
	Co-habiting	66 (88.0%)	9 (12.0%)	70 (93.9%)	5 (6.7%)
Education	No formal education	12 (100%)	0 (0.0%)	11 (91.7%)	1 (8.3%)
	Primary	16 (88.9%)	2 (11.1%)	16 (88.9%)	2 (11.1%)
	Junior high	91 (87.5%)	13 (12.5%)	88 (84.6%)	16 (15.4%)
	Senior high	426 (90.1%)	47 (9.9%)	420 (88.8%)	53 (11.2%)
	Vocational/technical/commercial	82 (83.7%)	16 (16.3%)	83 (84.7%)	15 (15.3%)
	Tertiary	259 (83.8%)	50 (16.2%)	263 (85.1%)	46 (14.9%)

**Table 5:** Recent practices by the socio-demographic background of participants.

Variable	Categories	Been to Crowded Places		*p*-Value	Travelled by Public Transport?		*p*-Value	Wash/Sanitized?		*p*-Value	Facemask-Wearing?		*p*-Value
		Yes	No		Yes	No		Yes	No		Yes	No	
Age	18–29	255 (58%)	185 (42%)	*p* = 0.70	202 (45.9%)	238 (54.1%)	*p* = 0.38	410 (93.2%)	30 (6.8%)	*p* = 0.77	421 (95.7%)	19 (4.3%)	*p* = 0.84
	30–39	295 (60.6%)	192 (39.4%)		229 (47.0%)	258 (53.0%)		448 (92.0%)	39 (8.0%)		464 (95.3%)	23 (4.7%)	
	40 and above	53 (60.9%)	34 (39.1%)		47 (54.0%)	40 (46.0%)		80 (92.0%)	7 (8.0%)		82 (94.3%)	5 (5.7%)	
Marital status	Never married	297 (55.4%)	239 (44.6%)	*p* = 0.02	231 (43.1%)	305 (56.9%)	*p* = 0.007	497 (92.7%)	39 (7.3%)	*p* = 0.66	511 (95.3%)	25 (4.7%)	*p* = 0.69
	Married	256 (63.5%)	147 (36.5%)		202 (50.1%)	201 (49.9%)		370 (91.8%)	33 (8.2%)		383 (95.0%)	20 (5.0%)	
	Co-habiting	50 (66.7%)	25 (33.3%)		45 (60.0%)	30 (40.0%)		71 (94.7%)	4 (5.3%)		73 (97.3%)	2 (2.7%)	
Education	No formal education	6 (50%)	6 (50%)	*p* = 0.69	10 (83.3%)	2 (16.7%)	*p* = 0.005	12 (100%)	0 (0.0%)	*p* = 0.03	12 (100%)	0 (0.0%)	*p* = 0.002
	Primary	13 (72.2%)	5 (27.8%)		14 (77.8%)	4 (22.2%)		15 (83.3%)	3 (16.7%)		14 (77.8%)	4 (22.2%)	
	Junior high	66 (63.5%)	38 (36.5%)		50 (48.1%)	55 (51.9%)		100 (96.2%)	4 (3.8%)		95 (91.3%)	9 (8.7%)	
	Senior high	284 (60%)	189 (40%)		206 (43.6%)	267 (56.4%)		443 (93.7%)	30 (6.3%)		457 (96.6%)	16 (3.4%)	
	Vocational/technical/commercial	56 (57.1%)	42 (42.9%)		51 (52.0%)	47 (48.0%)		93 (94.9%)	5 (5.1%)		93 (94.9%)	5 (5.1%)	
	Tertiary	178 (57.6%)	131 (42.4%)		147 (47.6%)	162 (52.4%)		275 (89.0%)	34 (11.0%)		296 (95.8%)	13 (4.2%)	

**Table 6:** Perception of vulnerability and wearing of face mask (N = 1014).

Variable	Categories	Vulnerability		Total	*p*-Value
		Vulnerable	Not Vulnerable		
Facemask use	Use	541(55.9%)	426(44.1%)	967 (100%)	<0.05
	Do not use	19(40.4%)	28(59.6%)	47 (100%)	

**Table 7:** Perceptions towards COVID-19 based on the health belief model.

Drivers	Perceived susceptibility	The second wave of COVID-19 infections was very scary
		Our job entails picking up total strangers whose health statuses are unknown to us
		Surface contamination of physical cash received greatly exposes us
		Passengers sometimes ignore you when you request them to sanitise their hands
	Perceived severity	I have not yet taken the COVID-1 vaccine, so I think I may fall severely sick
	Perceived benefits	I wear the facemask consistently to stay safe
		My employer insists on masking up, and I may lose my job if I do not comply
Barriers	Perceived barriers	None of my colleagues has so far fallen sick with COVID-19
		Only the elderly and people with underlying conditions are vulnerable, but we are young and healthy
		Facemask-wearing is challenging and uncomfortable
		Passengers mask up, so I do not feel the need also to wear the mask
		Local herbal preparations hold the cure even if one gets sick with COVID-19
		COVID-19 is not real

## Data Availability

The data presented in this study are available on request from the corresponding author.
